# An unusual occurrence of opsoclonus and liver enzymes elevation in a patient with acute motor and sensory axonal neuropathy subtype of Guillain-Barré syndrome

**DOI:** 10.1186/s12883-022-02599-0

**Published:** 2022-03-18

**Authors:** Hasan Alzuhaily, Eman khashaneh, Sanaa Albkhetan, Fatima Abbas

**Affiliations:** 1 Medical Student, Damascus University, Damascus, Syria; 2 Department of Neurology, Damascus University, Damascus, Syria; 3 Department of Internal Medicine, Damascus University, Damascus, Syria

**Keywords:** Guillain-Barré syndrome, Eye Movements, Liver Disease, Case Report, Opsoclonus

## Abstract

**Background:**

Acute motor and sensory axonal neuropathy (AMSAN) is a subtype of Guillain-Barré syndrome (GBS) differentiated by nerve conduction studies (NCS) and characterized by symmetric ascending paralysis often involving respiratory muscles. While opsoclonus, which is involuntary chaotic rapid eye movements, is not a common manifestation of GBS. Moreover, little published data are available on the relation between liver enzymes elevation and GBS.

**Case presentation:**

A 42-year-old man presented to Al Mouwassat University Hospital with weakness in all limbs and dyspnea. Examination showed an elevated respiratory rate, hyporeflexia, and decreased strength of upper and lower limbs. Analysis of cerebrospinal fluid revealed an albuminocyto-dissociation suggesting the diagnosis of GBS and subsequent plasmapheresis. NCS confirmed a diagnosis of AMSAN. Elevation in liver enzymes was noticed prompting further exploration with no positive findings. Despite treatment efforts, the patient developed severe dyspnea, deterioration in cognitive abilities, and opsoclonus with a normal brain MRI. Unfortunately, he developed respiratory failure which lead to his death.

**Conclusion:**

In this case, we highlight the occurrence of opsoclonus which is a rarely-encountered manifestation of GBS, in addition to an unexplained elevated liver enzyme, the thing that could contribute to larger research to further comprehend the pathophysiology of GBS.

**Supplementary Information:**

The online version contains supplementary material available at 10.1186/s12883-022-02599-0.

## Background

Guillain-Barré syndrome (GBS) is a neurological disorder characterized by often ascending paralysis clinically and albumino-cytological dissociation in the cerebrospinal fluid (CSF) analysis. The Main etiology is that a trigger induces the generation of autoantibodies targeting peripheral nerve myelin or axonal membranes, leading to specific electrophysiological features and significantly reduced conduction velocities [1]. GBS subtypes can be classified by clinical features and electrophysiological studies e.g. nerve conduction studies (NCS). Ascending paralysis and demyelination injury on NCS is called acute inflammatory demyelinating polyneuropathy; ascending paralysis, and reduced motor amplitudes but normal sensory amplitudes on NCS is called acute motor axonal neuropathy; profound paralysis, severe respiratory symptoms, and reduced or absent motor and sensory amplitudes is called acute motor and sensory axonal neuropathy (AMSAN). Other variants include Miller Fisher syndrome (MFS) characterized by ataxia, areflexia, and ophthalmoplegia, and Bickerstaff encephalitis characterized by hyperreflexia, encephalopathy in addition to features of MFS [2, 3]. Moreover, published cases reported different unusual presentations of GBS patients such as opsoclonus referring to a very rare disorder of eye movement characterized by involuntary chaotic rapid eye movement in all directions. It is mostly believed to be an autoimmune-mediated entity occurring as a para-infectious or paraneoplastic feature, or caused by toxic-metabolic causes, medications and remains unexplained in many cases [4, 5].

The exact pathogenesis of this phenomenon remains unclear, but it is proposed to be a result of either increased neuronal excitability in the paramedian pontine reticular formation (PPRF) which controls saccades or decreased omnipause neurons (OPNs) inhibition to the PPRF which results in ocular instability or oscillations [6].

Liver function disturbances in GBS patients are usually attributed to viral hepatitis with Cytomegalovirus (CMV), Epstein-Barr virus (EBV), hepatitis A (HAV), B (HBV), C (HCV), or E (HEV) virus. However, some GBS patients have disturbed liver functions with no concurrent infection [7]. Understanding these variants of GBS findings helps to establish a rapid diagnosis. In this case, we report an unusual presentation of opsoclonus and liver function disturbances in a GBS patient.

## Case presentation

A 42-year-old man presented to Al Mouwassat University Hospital with lower limb weakness that developed over the last 5 days followed by upper limbs weakness accompanied by paresthesia and numbness in upper and lower limbs. The patient was dyspneic at admission with New York Heart Association (NYHA) grade III (doing less than ordinary activity) [8]. No cognitive functions deterioration or seizures were mentioned. The review of other systems was unremarkable for any symptoms of preceding infection. Clinical examination revealed an elevated respiratory rate without affecting the patient’s ability to count to 20 on the single breath count test (SBCT), and a palpated liver span of 14 cm [9]. Neurological examination revealed hyporeflexia and decreased strength of upper and lower limbs with medical research council (MRC) sum-score of 36/60 [10]. Examination of Cranial nerves (CNs), sphincters function, deep sensations, and sensory innervation was normal.

Laboratory tests revealed an elevation in alanine transaminase (ALT) and aspartate transaminase (AST) on admission but normal other lab tests (Table 1).

**Table 1:** blood work and arterial blood gases of the patient upon admission. *WBC* white blood cells, *L/N* lymphocytes/Neutrophiles, *Hb* hemoglobin, *ESR* erythrocytes sedimentation rate, *LDH* lactate dehydrogenase, *CPK* creatinine phosphokinase, *TP* total protein, *ALB* albumin, *TB* total bilirubin, *DB* direct bilirubin, *ALP* alkaline phosphatase, *AST* aspartate aminotransferase, *ALT* alanine aminotransferase, *CRP* C—reactive protein, *PT* prothrombin time, *INR* international normalized ratio

WBC: 8400/mm3	TB: 1.13 mg/dl	Ca: 9.3 mg/dl	Blood gases:
L/N: 21/67	DB: 0.65 mg/dl	P: 4.1 mg/dl	Ph: 7.41
Hb: 14 g/dl	ALP: 111 U/L	PT: 91%	Po2: 67 mmHg
ESR: 17 mm\1 h	AST: 129 U/L	INR: 1	Pco2: 35 mmHg
CRP: 0.1 mg/dl	ALT: 374 U/L	TP:7.5 g/dl	HCO3: 22 mmol/l
LDH: 346 U/L	Na: 137 mEq/L	ALB: 4.5 g/dl	SatO2:93%
CPK: 21 U/L	K: 3.9 mmol/l		

Analysis of cerebrospinal fluid revealed an albuminocyto-dissociation with WBCs of 4 /mm^3^, elevated total protein of (92 mg/dl), and a normal glucose level.

Summarizing clinical symptoms and CSF analysis suggested the diagnosis of GBS and implicated plasmapheresis as a modality of treatment. However, elevated liver enzymes indicated further exploration. Echography of the liver and biliary tract revealed a 14 cm liver span and grade 1 fatty liver changes with no dilation of biliary tracts in and out of the liver, the thing that cannot by itself explain serum findings. Serology tests for viral hepatitis with HBV and HCV were negative. Toxicology screening and porphyria investigation were also negative.

NCS demonstrated no sensory nor motor response after stimulating lower limbs nerves and F wave was absent. There was also a significant decrease in motor and sensory responses amplitude and absence F wave in upper limbs. These findings proved severe motor and sensory axonal injury confirming a diagnosis of AMSAN.

**Fig. 1:**
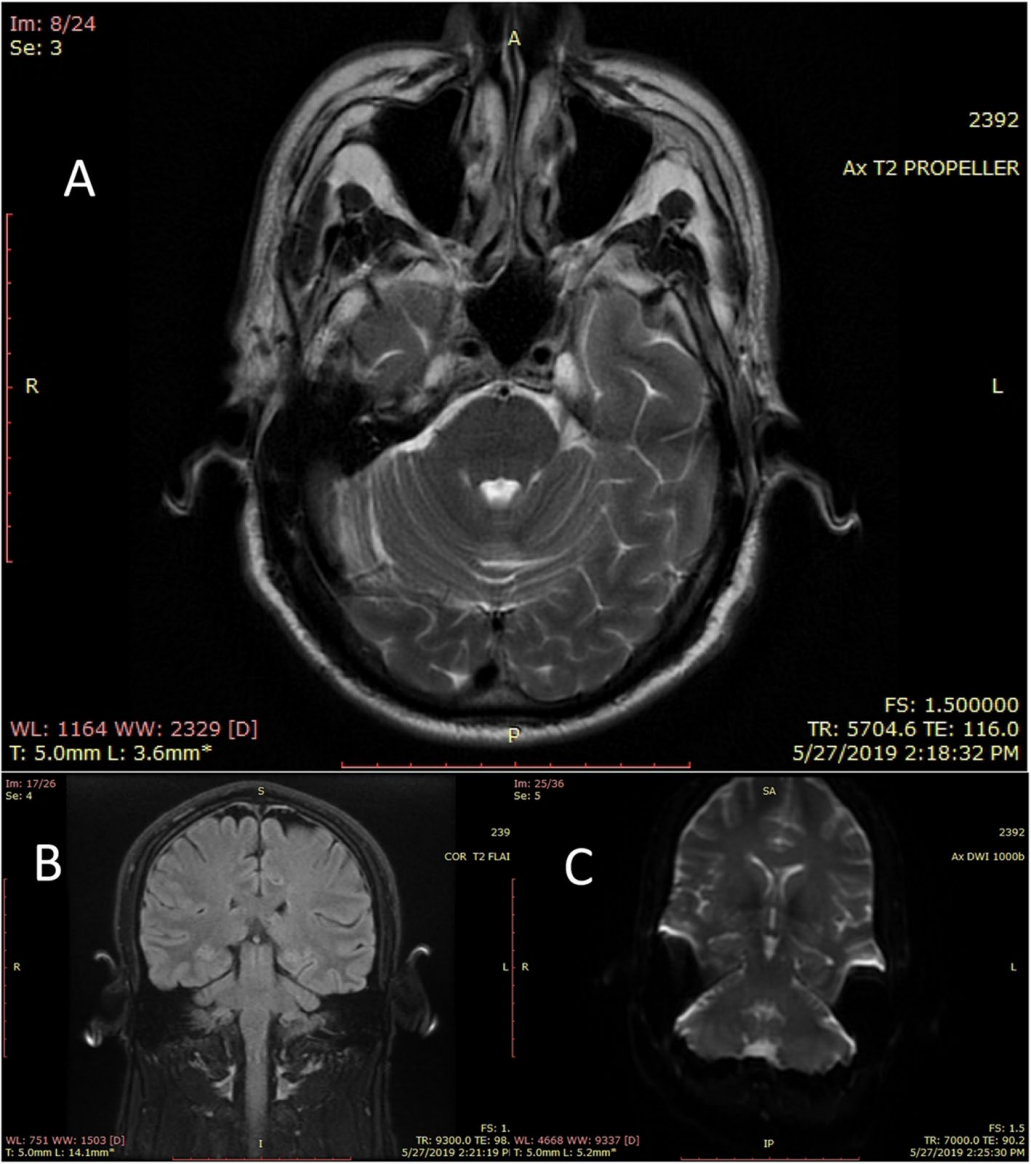
Sections of the MRI images of the patient at the brainstem level. **A**. T2 axial section. **B**. T2 flair coronal section. **C**. diffusion-weighted coronal section

Despite following therapeutic plasmapheresis for five sessions, the patient developed severe dyspnea (grade IV at NYHA scale), an SBCT of 10, and a decrease in motor strength in upper and lower limbs (MRC sum-score of 18) followed by CN 7 bilateral palsy. Concomitantly, the neurological exam revealed a deterioration in cognitive abilities with visual hallucinations, disorientation to people, and opsoclonus (showed in an additional movie file [see Additional file 1]) that was managed by clonazepam. Therefore; an MRI (Magnetic Resonance Imaging) was performed with normal findings (Fig. 1 [see also additional file 2, additional file 3, and additional file 4 for the whole MRI series]), laboratory tests showed a progressive elevation in liver enzymes. After that, the patient developed a sputum-producing cough with bilateral basal consolidations on the chest X-ray suggesting nosocomial pneumonia. Later, the patient developed a severe respiratory failure resulting in a cardio-respiratory arrest despite the administration of intravenous antibiotics and mechanical ventilation. Regardless of the resuscitation trials, the respiratory failure resulting from both severe muscle involvement and infection led to his death.

## Discussion and conclusion

To our knowledge, clinical variants of GBS in the Syrian population have not been described in the literature which highlights the importance of reporting clinical extraordinary presentations. Our patient’s findings fulfilled the diagnostic criteria of GBS; the patient had a severe ascending weakness with hyporeflexia and bilateral facial nerve palsy and opsoclonus with NCS revealing the absence of motor and sensory response in addition to CSF test results. He didn’t have ataxia or ophthalmoplegia nor hyperreflexia or encephalopathy on admission the thing that shifted the diagnoses away from miller fisher syndrome and Bickerstaff encephalitis respectively. However, Bickerstaff encephalitis couldn’t be fully excluded despite the normal MRI imaging and due to lack of performance of GQ1b antibody screening. According to the few published data regarding opsoclonus in GBS patients, the pathophysiology of this clinical variant remains unexplained. However, multiple studies are suggesting an association between opsoclonus and immunological diseases with elevated antibodies titers which could account for the association between opsoclonus and GBS [6, 4]. It could be attributed to the common autoimmune nature of both diseases. This may also support that GBS syndrome may affect CNS, based on the theory of CNS dysfunction (disruption of OPN or excitation of PPRF) causing opsoclonus. However, we did not manage to perform GQ1b nor paraneoplastic antibody screening to determine a possible paraneoplastic trigger for opsoclonus. The occurrence of opsoclonus with GBS is reported in one case in the literature, but unlike in our case, the patient improved and got well with resolved symptoms including the opsoclonus [5].

From another point, a published study suggested that liver function disturbances are present in up to 56% of GBS patients on admission [11]. Another prospective study on GBS patients found that 38% of patients had elevated liver enzymes of which 28% tested negative for known causes of liver damage [7]. Other studies reported liver enzymes elevation in GBS patients with concurrent viral hepatitis with hepatitis A, B, C or E or CMV or EBV, or autoimmune hepatitis [12, 13, 14]. However, some cases of GBS cause Liver function disturbances without concurrent infections [15, 7]. Although elevated liver enzymes in GBS patients are studied as a risk factor for a worse prognosis and the need for mechanical ventilation, the specific cause of this finding is not well-studied [16, 17]. It may be a hepatic manifestation of the autoimmune process occurring in GBS in general or in the AMSAN subtype in particular.

Our patient was negative for hepatitis B and C viruses and porphyria, with a clear toxicology screen and a negative history of alcoholism. Although HAV, HEV, CMV, EBV, and autoimmune hepatitis work up were planned, we did not get to complete the exploration for liver functions disturbances. Isolated ALT and AST elevation without hypoalbuminemia or prolonged prothrombin time suggested no liver failure. Administration of antibiotics to treat pneumonia, and clonazepam to treat the opsoclonus and the continuous deterioration of the patient neurological status decreased the cost-effectiveness of pursuing liver study leaving the etiology unclear and possibly attributed to either untested viral infection or due to the GBS autoimmune process itself.

In conclusion, our case presents a severe form of GBS case with unique laboratories and clinical manifestation that might suggest new theories to be tested in more profound and bigger studies.

We encourage similar reporting of cases to further understand the mechanisms and relations between these entities to improve diagnostic approaches and prognostic evaluation to such patients and avoid unfortunate results.

## Supplementary Information

**Additional file 1. **

**Additional file 2. **

**Additional file 3. **

**Additional file 4. **

## Data Availability

Not applicable.

## Abbreviations

ALT: Alanine transaminase
AMSAN: Acute motor and sensory axonal neuropathy
AST: Aspartate transaminase
CMV: Cytomegalovirus
CNs: Cranial nerves
CSF: Cerebrospinal fluid
EBV: Epstein-Barr virus
GBS: Guillain-Barré syndrome
HAV: Hepatitis A virus
HBV: Hepatitis B virus
HCV: Hepatitis C virus
HEV: Hepatitis E virus
MFS: Miller Fisher syndrome
MRC: Medical Research Council
MRI: Magnetic resonance imaging
NCS: Nerve conduction studies
NYHA: New York Heart Association
OPNs: Omnipause neurons
PPRF: Paramedian pontine reticular formation
SBCT: Single breath count test
