# Supplementary figures and images for: An unusual occurrence of opsoclonus and liver enzymes elevation in a patient with acute motor and sensory axonal neuropathy subtype of Guillain-Barré syndrome
Source: BMC Neurol. 2022 Mar 18;22:102. doi: 10.1186/s12883-022-02599-0 (PMC8932169; doi:10.1186/s12883-022-02599-0)

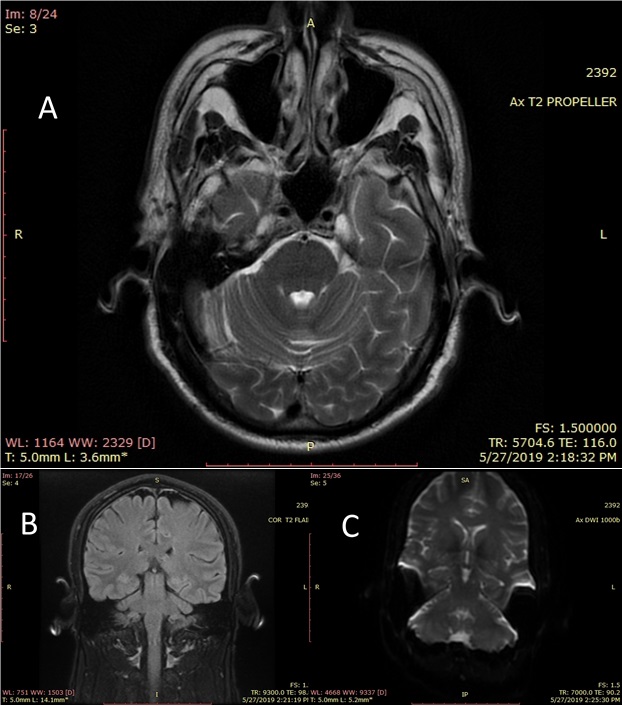

Supplement: Supplementary file 2 — Additional file 2. [file 12883_2022_2599_MOESM2_ESM.jpeg]
